# Shared Decision Aids: Increasing Patient Acceptance of Long-Acting Reversible Contraception

**DOI:** 10.3390/healthcare3020205

**Published:** 2015-04-10

**Authors:** Tracy P. George, Claire DeCristofaro, Bonnie P. Dumas, Pamela F. Murphy

**Affiliations:** 1 Department of Nursing, Francis Marion University, Florence, SC 29502, USA; E-Mail: TGeorge@fmarion.edu; 2 South Carolina Department of Health and Environmental Control, Dillon, SC 29536, USA; 3 College of Nursing, Medical University of South Carolina, Charleston, SC 29425, USA; E-Mail: dumasb@musc.edu; 4 Department of Behavioral Sciences, College of Health, Human Services, and Science, Ashford University, San Diego, CA 92123, USA; E-Mail: pamela.murphy@ashford.edu

**Keywords:** long-acting reversible contraceptive methods (LARCs), shared decision aids, intrauterine devices (IUDs), contraceptive implants, patient education, public health nursing

## Abstract

Unintended pregnancies are an important public health issue. Long-acting reversible contraceptive methods (LARCs) are reliable, safe, highly effective methods for most women; however they are underutilized in the United States. Shared decision aids were added to usual care in five public health family planning clinics in the Southeastern United States, staffed by advance practice nurses and registered nurses. All five sites showed an increase in the use of LARCs during the time period that shared decision aids were used (results statistically significant to *p* < 0.001). It is important for women to make informed choices about contraception, and shared decision aids can be utilized to support this decision making. This resource has been adopted for statewide use in all public health clinics, and implications for practice suggest that the use of shared decision aids is an effective method to support informed patient decision making and acceptance of LARC methods of contraception.

## 1. Introduction

It is important for women to make informed decisions about contraception without bias or coercion. Shared decision-making is a patient education approach that acknowledges the patient’s preferences, allows the patient to make informed choices, shows respect for the patient’s decisions, and is associated with improved satisfaction with healthcare decisions [1]. In addition, shared decision-making is often related to improved health outcomes [2]. Shared decision-making involves the patient and provider working together to reach a healthcare decision [3]; through the use of shared decision aids, patients are able to comprehend their choices and possible outcomes and participate more effectively in health care decision-making [4]. In this way, shared decision aids are tools that assist with the process of shared decision making.

The use of shared decision aids is prevalent in many areas of health care, and may include brochures, videos, or internet-based activities; they may be self- or provider-administered [5]. The use of shared decision aids regarding contraceptive choices has been associated with improved communication and more informed decision-making [6]. Currently, the Agency for Healthcare Research and Quality (AHRQ) provides patient-centered shared decision aid resources, such as the “SHARE” stepwise approach that includes patients in healthcare decision making by helping the patient compare options and evaluating patient preferences [7].

Unintended pregnancies are an important issue in health care today. Nearly half of all pregnancies in the United States are unintended, and the direct health care costs of those pregnancies are estimated to be $9.6 to 12.6 billion annually [8]. Long-acting reversible contraceptive methods (LARCs), which include intrauterine devices (IUDs) and contraceptive implants, are highly effective types of reversible contraception, and limited effort is needed by the user for adherence. According to the Centers for Disease Control (U.S. Medical Eligibility Criteria for Contraceptive Use, 2010) and endorsed by the American College of Obstetrics and Gynecology (ACOG), there are few limitations or contraindications to the use of LARCs [9,10]. While the initial expense is higher, LARCs are also very cost-effective contraceptive methods [11]. Despite the positive benefits of LARCs, their use remains low. In the United States, IUD usage has grown from 0.8% to 5.6% from 1995 to the 2006–2010 time period [12]. Globally, contraceptive implants and contraceptive injections are used by 3.4% of women, while 15.5% of women utilize IUDs [13]. Improved patient education, removal of financial barriers, and increased provider knowledge and training are necessary to expand the use of LARCs. As per ACOG, increased utilization of LARCs could reduce unintended pregnancy rates in the United States [10]. 

Contraceptive knowledge is an issue that related to LARC usage by women. Dempsey, Billingsley, Savage, and Korte [14] found that women who have increased knowledge about LARCs are more likely to choose them for contraception. In addition, women who receive effective counseling that includes the benefits and potential side effects of LARCs, are more likely to be satisfied with and continue these methods [11]. Furthermore, eighty-five percent of private and public sector family planning providers identified a need for improved contraceptive counseling to enhance contraceptive method utilization [15].

Arrowsmith, Aicken, Saxena, and Majeed [16] completed a systematic review of the literature on ways to increase the use of the copper IUD. Three studies reported that community-based contraceptive counseling and referrals resulted in increased use of the non-hormonal copper IUD (OR 2.0, 95% CI 1.4 to 2.85). Two studies on antenatal counseling and uptake of the copper IUD were statistically significant (OR 2.33, 95% CI 1.39 to 3.91). In one study, postpartum contraceptive counseling resulted in more women choosing the non-hormonal IUD (OR 5.73, 95% CI 3.59 to 9.15).

Contraceptive counseling was also an important factor in several other studies. In a qualitative study of 20 nulliparous young women, using semi structured 1-h interviews, Brown, Auerswald, Eyre, Deardorff, and Dehlendorf [17] developed a process model for intrauterine contraception that focuses on the role of the provider. This model includes the following stages: initial awareness, initial reaction, information gathering, adoption, adjustment, and reassessment. Patients emphasized the importance of receiving comprehensive contraceptive counseling when making the decision to choose LARCs. In a cross-sectional descriptive survey of 1800 unmarried males and females, Dempsey *et al.* [14] found that patients with high IUD knowledge were six times more likely to be using a LARC (OR 6.3, 95% CI 1.4–28.8).

In a prospective cohort study of 7637 women, Madden, Mullersman, Omvig, Secura, and Peipert [18] found that 78% of women at a community partner clinic who received the usual contraceptive counseling chose LARCs, while 72% at the university clinic who received structured contraceptive counseling provided by non-medical staff members decided to use LARCs. The differences between the groups were not significant (adjusted relative risk 0.98, 95% CI 0.94–1.02). In a prospective cohort study of 2500 women, Secura, Allsworth, Madden, Mullersman, and Peipert [19] found that 67% of women who were not on a contraceptive method chose LARCs once the barriers of patient education and cost were removed. Of those using LARCS, 56% chose intrauterine contraception, while 11% chose the contraceptive implant. In a prospective cohort study of 7486 women who were offered contraceptives of their choice at no cost in the Contraceptive CHOICE Project, the failure rate for contraceptive injections and LARCs was 0.27 per 100 participant years, regardless of whether the person was under 21 years of age or over 21 years of age [20]. In comparison, participants who chose contraceptive rings, pills, or patches had a failure rate of 4.55 per 100 participant years, with higher rates in women under age 21 [20]. Among 1404 adolescents involved in the Contraceptive CHOICE prospective cohort study, the teen birth rate was decreased to 34.0 per 1000, while the national teen pregnancy rate was 57.4 per 1000 teens. There were similar reductions in live births and induced abortion rates among the study participants (19.4 and 19.7 per 1000), as compared to national data (94.0 and 41.5 per 1000) [21].

In a qualitative study of 42 female patients, most patients desired a patient-centered approach to counseling, rather than a directive approach. Dehlendorf, Levy, Kelley, Grumbach, and Steinauer [22] found that patients desired to make the final decisions about contraception with input from providers. Patients also stated the need for verbal and written information on contraceptives, and they emphasized the role of social networks in their decisions about contraceptive methods. 

According to the American College of Obstetrics and Gynecology (ACOG) Committee Opinion, providers should discuss LARCs as first-line contraceptive options for many women [10]. Comprehensive contraceptive counseling is an important factor in the adoption of LARCs by females of reproductive age. Many individuals are unaware of LARCs [23] unless health care providers present information about them, so it is important for advanced practice nurses to discuss LARCs as a contraceptive option in 2013, the counties studied in this project had low utilization of LARCs. In the three counties of the project, 1.63% to 2.67% of public health family planning females had IUDs, while 1.27% to 3.67% of the patients had contraceptive implants [24]. Furthermore, in 2012, the teen birth rates for the three counties were above the national average of 29.4 births per 1000 females, with a range of 35.1 to 67.1 births per 1000 females in the three counties [25]. In this public healthcare setting, nursing staff used shared decision aids in addition to regular care for three months to determine if the use of shared decision aids impacted the number of LARCs inserted over that time period.

## 2. Experimental Section

### 2.1. Project Design

This was a six-month pre-intervention/post-intervention quality improvement project at five public health family planning sites in three counties in the southeastern United States. Usual care of females, ages 13–45 years old, seeking contraception included verbal counseling by staff (nurses and nurse practitioners) as part of the review of consent documentation regarding contraceptive options. Shared decision aid brochures were added to usual care by the regular staff, and the number of LARCs inserted for three months prior to the intervention was compared with the number of LARCs inserted during the three-month intervention period.

Most of the public health nurses in the five sites have completed additional training to expand the general skill-set to include advanced assessment and performance of physical examinations skills. Integrated Preventative Health RNs provide contraceptives under standing orders and additionally are authorized by the state’s Nurse Practice Act to practice in an expanded role. The nurse practitioners are either family nurse practitioners or women’s health nurse practitioners, and in addition to performing exams, they are all trained to insert IUDs and contraceptive implants at the sites. Nurse practitioners are available at the sites at least one day per week. 

### 2.2. Institutional Review Board Approval

The Institutional Review Board for the state health department, which is the administrative body for the family planning program, approved this project prior to implementation.

### 2.3. Development and Implementation of Shared Decision Aids

Guidance for nurses’ usual care when counseling females of reproductive ability on contraceptive options was provided by the current version of Title X program guidelines, published by the United States Department of Health and Human Services [26]. In addition, the five clinics shared the same public governing agency, which has a policy on patient education that guides contraceptive counseling [27]. Adhering to this policy, the nurses and nurse practitioners utilize a collaborative approach to counseling patients, which is consistent with shared decision making. As per agency guidelines, all written literature must be appropriate for the person’s age, educational level, language, and sociocultural backgrounds [27].

**Figure 1:**
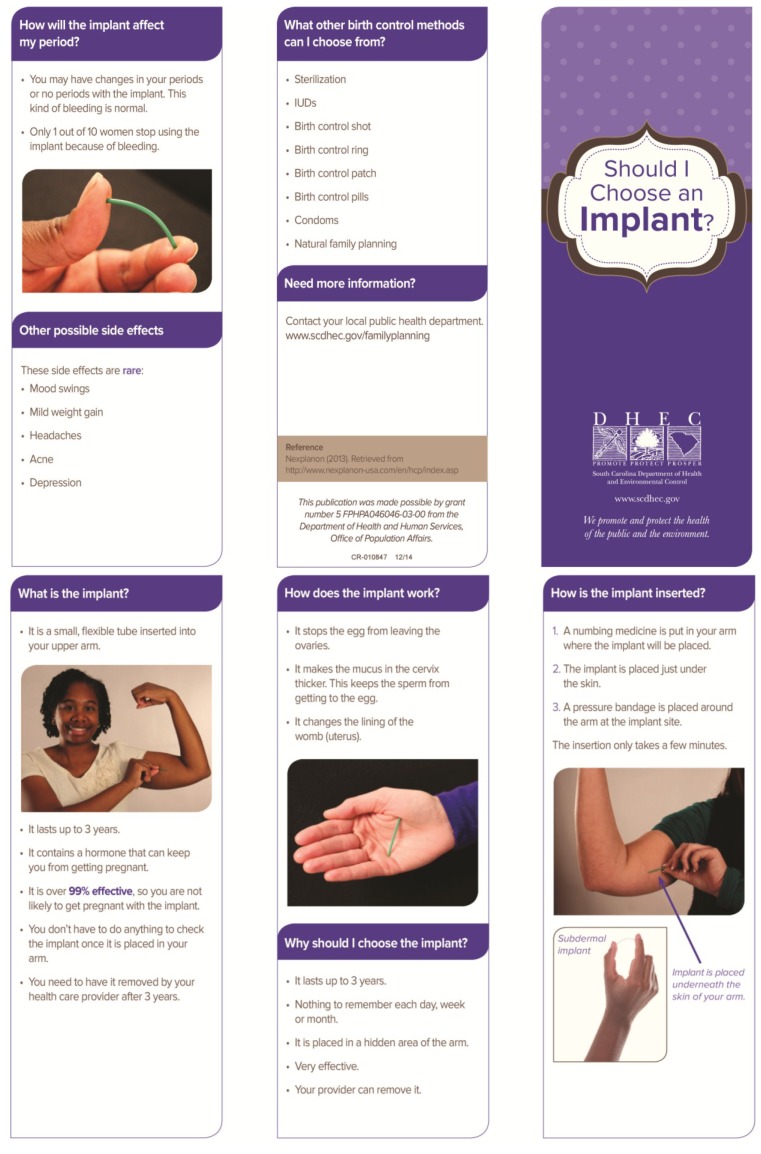
Shared Decision Aid, English Language: “Should I Choose an Implant?” [28].

**Figure 2:**
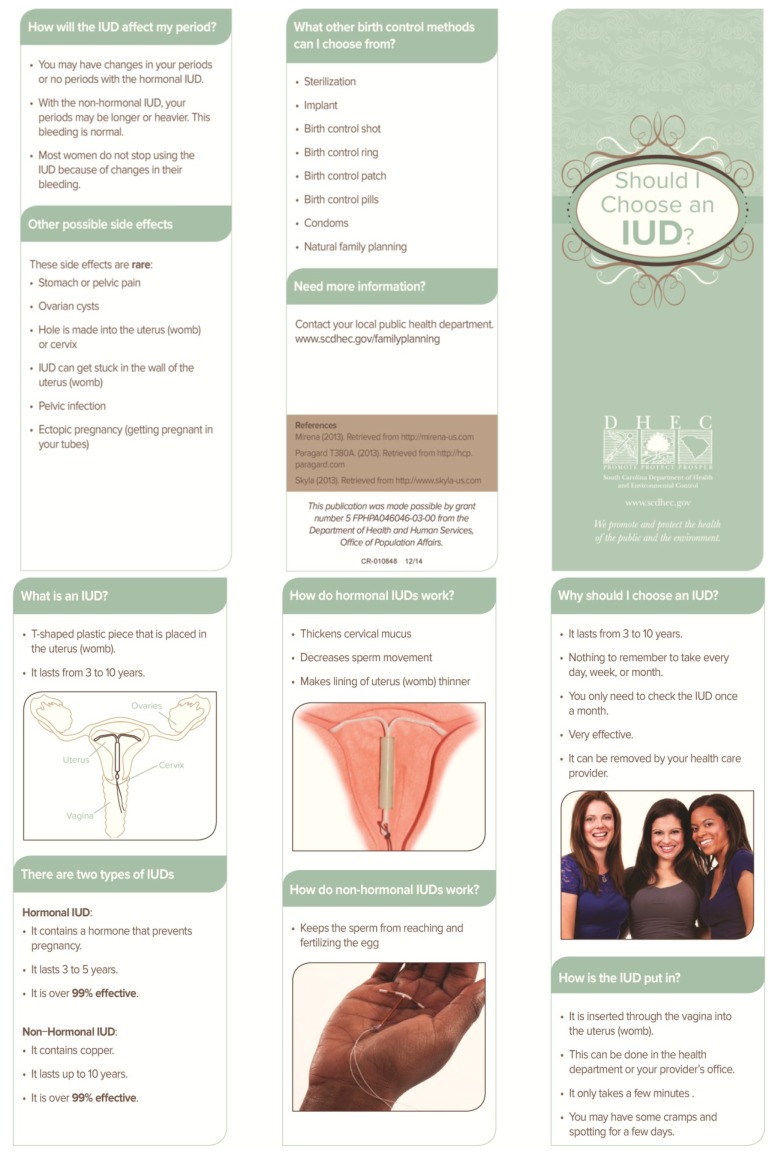
Shared Decision Aid, English Language: “Should I Choose an IUD?” [29].

**Figure 3:**
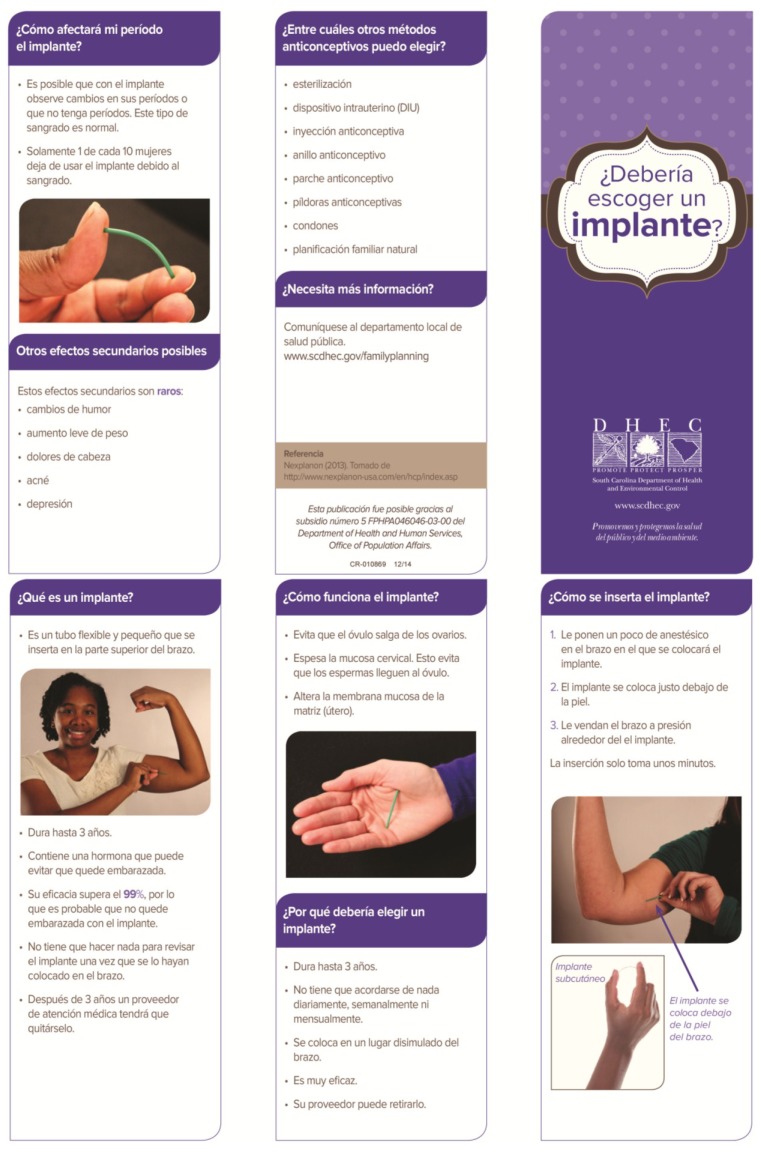
Shared Decision Aid, Spanish Language: “¿Debería escoger un implante?” [30].

**Figure 4:**
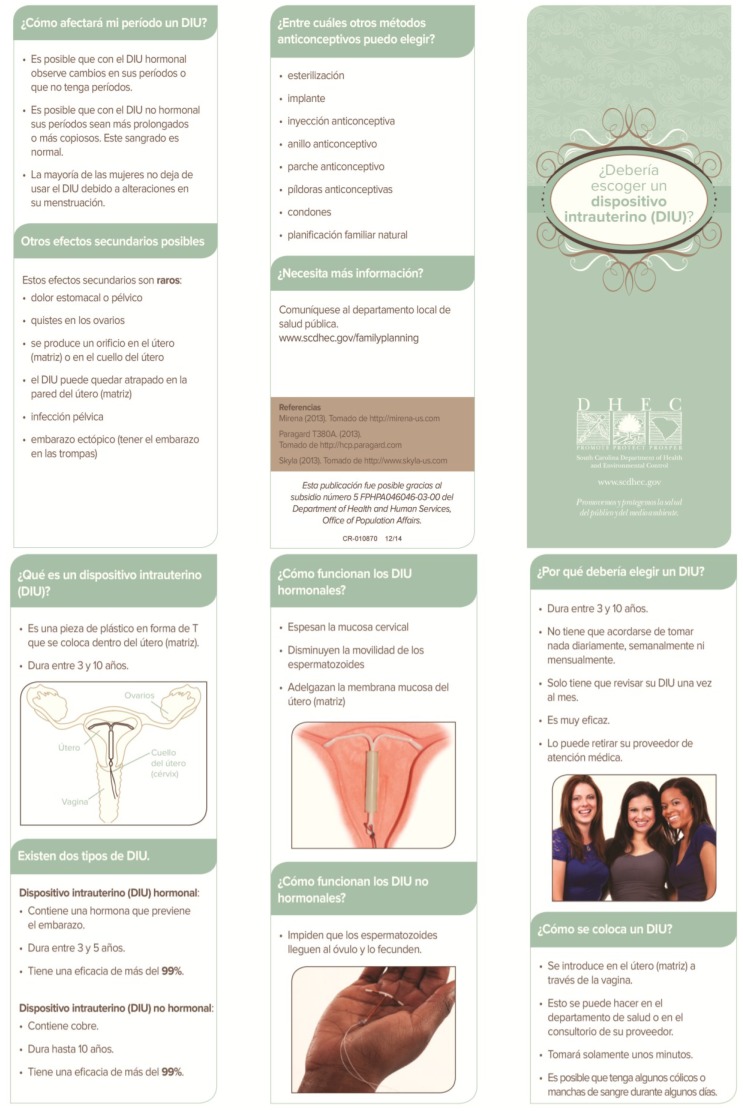
Shared Decision Aid, Spanish Language: “¿Debería escoger un dispositivo intrauterino(DIU)?”[31].

Prior to the project, a one-page sheet describing all the contraceptive methods was utilized in counseling patients. Women were allowed additional information on any contraceptive methods. The two shared decision aids used for this project were designed by one of the project authors (TPG), in conjunction with local, regional, and state agency personnel, as well as input from community members and public health patients. The shared decision aids are color, trifold brochures that utilize pictures and bullet points, and each of these were made available in both English and Spanish language versions (translation provided by professional translators employed by the state) (See Figure 1, Figure 2, Figure 3 and Figure 4). 

In April 2014 a brief educational intervention was provided to the staff nurses and nurse practitioners about the shared decision aids, prior to their use in the five clinics. The nurses and nurse practitioners viewed a 15-min evidence-based presentation on LARCs, shared decision aids, and the practice improvement project itself; the presentation was designed by author TPG. Nursing staff attendance was verified by the nursing site supervisors, and there was 100% compliance in the training. The shared decision aids were added to routine care, used in a non-directive, collaborative approach appropriate to shared decision making. The nurses and nurse practitioners reviewed LARC choices with patients during patient encounters, utilizing the shared decision aids in the clinic setting to assist with responding to patient questions and clarifying information about procedures. Patients could also bring the shared decision aids home if desired to review further before selecting their chosen method of contraception.

## 3. Results and Discussion

**Figure 5:**
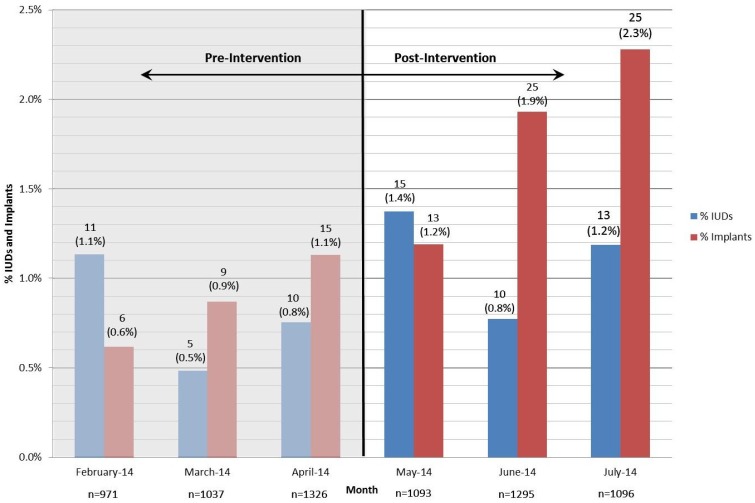
Summary Graph for LARCs Inserted at Five Public Health Sites.

During the study, 3484 women were approached. The total number of pre-intervention patients receiving contraceptive counseling for the months of 18 February through April 2014 (no shared decision aids) *versus* post-intervention data May through 19 July 2014 (use of shared decision aids) is compiled in Table 1. The total number of LARC insertions increased from 56 LARCs pre-intervention (*n* = 56, 1.7%) to 101 LARCs post-intervention (*n* = 101, 2.9%) (see Figure 5). A chi-square test of independence comparing the pre-intervention (no shared decision aid) and post-intervention (use of shared decision aid) for utilization of LARCs showed statistically significant results (χ^2^ = 12.23 with 1 degree of freedom, *p* < 0.005).

The utilization of contraceptive implants increased from 30 insertions pre-intervention to 63 insertions post-intervention, which corresponds to an increase from 0.9% (*n* = 30) prior to the intervention to 1.8% (*n* = 63) following the intervention. The number of IUDs grew from 26 IUDs pre-intervention to 38 IUDs post-intervention, which is a slight increase from 0.8% (*n* = 26) to 1.1% (*n* = 38) of total patients. The IUDs utilized were the non-hormonal IUDs and hormonal Mirena IUDs. The Mirena IUDs were inserted the majority of the time; only 5 Paragard IUDs were inserted during the study. A chi square test of independence comparing the choice of IUD *versus* implants was not statistically significant (χ^2^ = 1.15 with 1 degree of freedom, *p* > 0.25). In addition, no patients had the LARCs removed during the study period.

**Table 1:** Total Number of Implants and IUDs Inserted at Five Public Health Sites.

	Site 1	Site 2	Site 3	Site 4	Site 5	Total
Pre-intervention	n_pre_ = 700	n_pre_ = 495	n_pre_ = 382	n_pre_ = 1077	n_pre_ = 680	n_pre_ = 3334
Implants	12 (1.7%)	5 (1.0%)	0 (0%)	6 (0.6%)	7 (1.0%)	30 (0.9%)
IUDs	1 (0.1%)	1 (0.2%)	3 (0.8%)	16 (1.5%)	5 (0.7%)	26 (0.8%)
Total LARCs	13 (1.9%)	6 (1.2%)	3 (0.8%)	22 (2.0%)	12 (1.8%)	56 (1.7%)
Post-intervention	n_post_ = 691	n_post_ = 509	n_post_ = 504	n_post_ = 1100	n_post_ = 680	n_post_ = 3484
Implants	15 (2.2%)	12 (2.4%)	4 (0.8%)	17 (1.6%)	15 (2.2%)	63 (1.8%)
IUDs	4 (0.6%)	4 (0.8%)	3 (0.6%)	20 (1.8%)	7 (1.0%)	38 (1.1%)
Total LARCs	19 (2.8%)	16 (3.2%)	7 (1.4%)	37 (3.4%)	22 (3.2%)	101 (2.9%)

n_pre_: n pre-intervention; n_post_: n post-intervention.

Informal feedback from patients and staff occurred spontaneously during the course of this project. Many patients provided positive statements on use of the shared decision aids for LARCs, including that they “liked the pictures” and the decision aids were “easy to understand.” The nurses and nurse practitioners had a similar positive experience, stating that the shared decisions aids were “helpful in counseling patients” and “allowed patients to make more informed decisions”.

The clinics do not have nurse practitioner availability on each day of the week, so that is a barrier to same-day insertions. The clinics provide LARCS on a sliding-fee scale, and they also accept private insurance plans and all forms of Medicaid. The clinics have LARCS stocked and easily available for insertions. The clinics have adopted quick starts to LARCs when nurse practitioners are on site. IUDs can be inserted when patients are not on their menses, and STD testing can be performed at the time of insertion.

The combined five sites showed an increase in utilization of LARCs during the time period that shared decision aids were used, with the greatest increase in LARCs coming from the use of contraceptive implants. Informally, implementation was well accepted by providers, staff and patients. These results support the use of shared decision aids, which may provide patients with more information, thus likely improving women’s ability to make more knowledgeable decisions regarding LARCs. This resource has been adopted for statewide use in all public health clinics.

Strengths of the project included the positive agency and nursing staff and nursing provider support for the quality improvement project. Once the shared decision aids were developed and approved, the costs to the sites were minimal since the shared decision aids were provided to all clinic sites at no cost; implementation of this project only required obtaining printed decision aid brochures for distribution to patients, and a brief educational intervention for staff. In addition, the use of the shared decision aids did not place any additional time demands on the nursing staff or nurse practitioner providers.

A limitation of the project included a short post-implementation time frame of 3 months. A longer project might provide more robust data to better demonstrate changes in the percentage of LARC insertions. There are small numbers of overall of LARCs inserted, and staffing accounts for some of the variability between locations. Nurse practitioner availability for performing procedures is another factor that may influence LARC insertion rates, since some of the clinic locations have a nurse practitioner only once a week or less. The sites with more nurse practitioner access could provide same-day insertions, while the sites with clinicians less frequently had to schedule insertions. In addition, the baseline knowledge of LARCS was not obtained. This project only considers the variable of introducing shared decision making via the use of printed decision aids. However, many factors may influence women’s choice of contraceptive methods, including the opinions of friends, partners, family, and information from the media [32]. In addition, costs, side effects, confidentiality of care, knowledge about methods, perceived risk of pregnancy, use of alcohol, and lack of planning affect contraceptive decisions [33].

The higher use of implants compared to IUDs may be related to patient preference and or increased clinician comfort with implants. Further studies need to be done to determine the reasons for the differences in types of LARCs inserted.

The utilization of LARCs in this project increased from 1.9% to 2.7%. However, this is much lower than United States and global utilization of LARCs. In the United States from 2006 to 2010, 5.6% of women used IUDs [26], while 3.4% of women worldwide used contraceptive implants and contraceptive injections and 15.5% of women utilized IUDs. There is a need to consider ways to remove barriers to LARC usage in this setting, including increased provider availability for same-day LARC insertions.

## 4. Conclusions

Our project provides evidence that the use of shared decision aids was an intervention that may have improved the use of LARCs at the five public health sites. Further studies need to investigate potential benefits from including shared decision aids in regular care for other types of healthcare services, both in the public and private setting. If the benefits of using shared decision aids are validated by such research, we advocate for making their use part of the standard of care.
